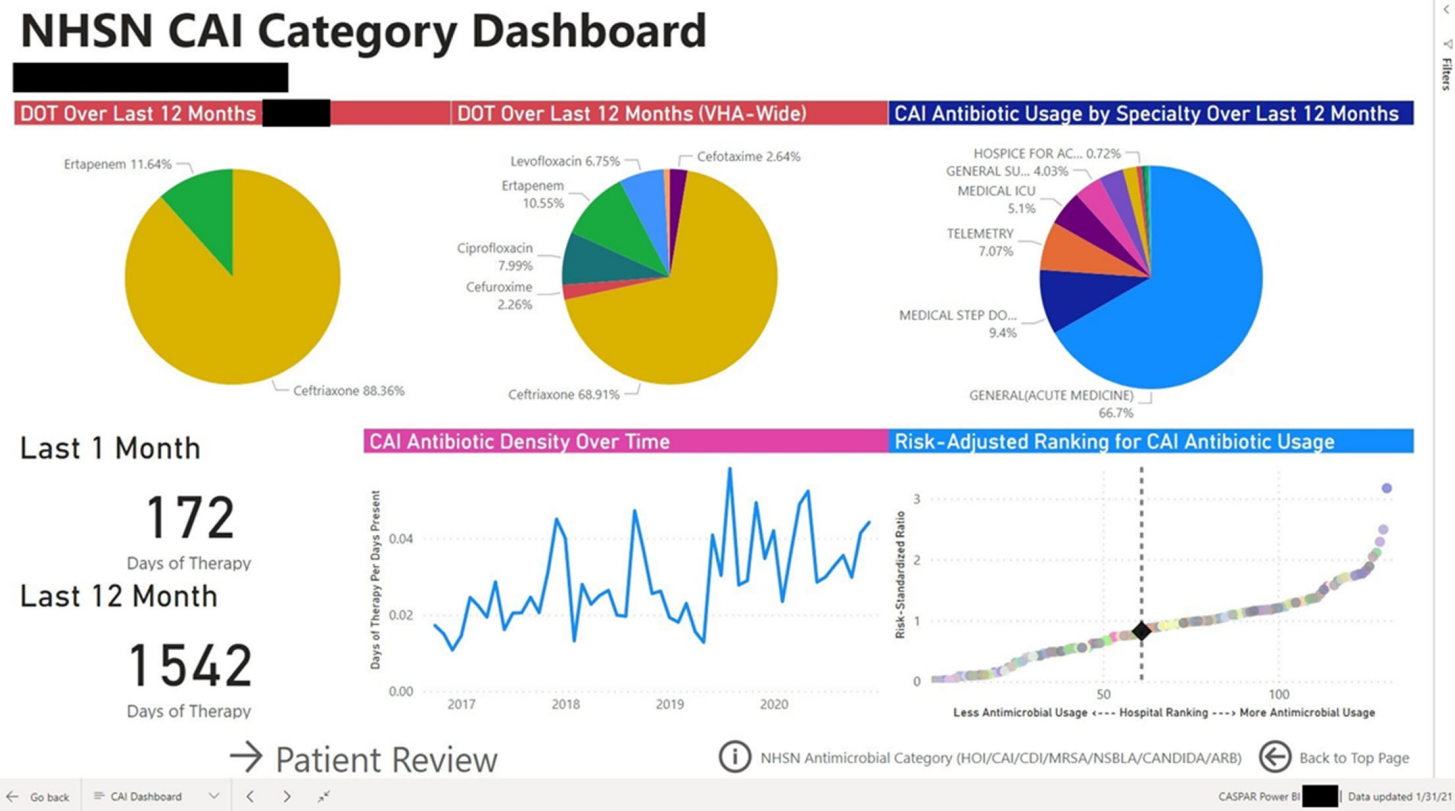# Automated Nationwide Benchmarking Dashboard for Antimicrobial Stewardship Programs within the Veterans’ Health Administration

**DOI:** 10.1017/ash.2021.43

**Published:** 2021-07-29

**Authors:** Michihiko Goto, Eli Perencevich, Alexandre Marra, Bruce Alexander, Brice Beck, Daniel Livorsi, Julia Friberg, Christopher Richards, DeShauna Jones, Michael Sauder

## Abstract

**Group Name:** VHA Center for Antimicrobial Stewardship and Prevention of Antimicrobial Resistance (CASPAR) **Background:** Antimicrobial stewardship programs (ASPs) are advised to measure antimicrobial consumption as a metric for audit and feedback. However, most ASPs lack the tools necessary for appropriate risk adjustment and standardized data collection, which are critical for peer-program benchmarking. We created a system that automatically extracts antimicrobial use data and patient-level factors for risk-adjustment and a dashboard to present risk-adjusted benchmarking metrics for ASP within the Veterans’ Health Administration (VHA). **Methods:** We built a system to extract patient-level data for antimicrobial use, procedures, demographics, and comorbidities for acute inpatient and long-term care units at all VHA hospitals utilizing the VHA’s Corporate Data Warehouse (CDW). We built baseline negative binomial regression models to perform risk-adjustments based on patient- and unit-level factors using records dated between October 2016 and September 2018. These models were then leveraged both retrospectively and prospectively to calculate observed-to-expected ratios of antimicrobial use for each hospital and for specific units within each hospital. Data transformation and applications of risk-adjustment models were automatically performed within the CDW database server, followed by monthly scheduled data transfer from the CDW to the Microsoft Power BI server for interactive data visualization. Frontline antimicrobial stewards at 10 VHA hospitals participated in the project as pilot users. **Results:** Separate baseline risk-adjustment models to predict days of therapy (DOT) for all antibacterial agents were created for acute-care and long-term care units based on 15,941,972 patient days and 3,011,788 DOT between October 2016 and September 2018 at 134 VHA hospitals. Risk adjustment models include month, unit types (eg, intensive care unit [ICU] vs non-ICU for acute care), specialty, age, gender, comorbidities (50 and 30 factors for acute care and long-term care, respectively), and preceding procedures (45 and 24 procedures for acute care and long-term care, respectively). We created additional models for each antimicrobial category based on National Healthcare Safety Network definitions. For each hospital, risk-adjusted benchmarking metrics and a monthly ranking within the VHA system were visualized and presented to end users through the dashboard (an example screenshot in Figure 1). **Conclusions:** Developing an automated surveillance system for antimicrobial consumption and risk-adjustment benchmarking using an electronic medical record data warehouse is feasible and can potentially provide valuable tools for ASPs, especially at hospitals with no or limited local informatics expertise. Future efforts will evaluate the effectiveness of dashboards in these settings.

**Funding:** No

**Disclosures:** None

**Figure 1.**
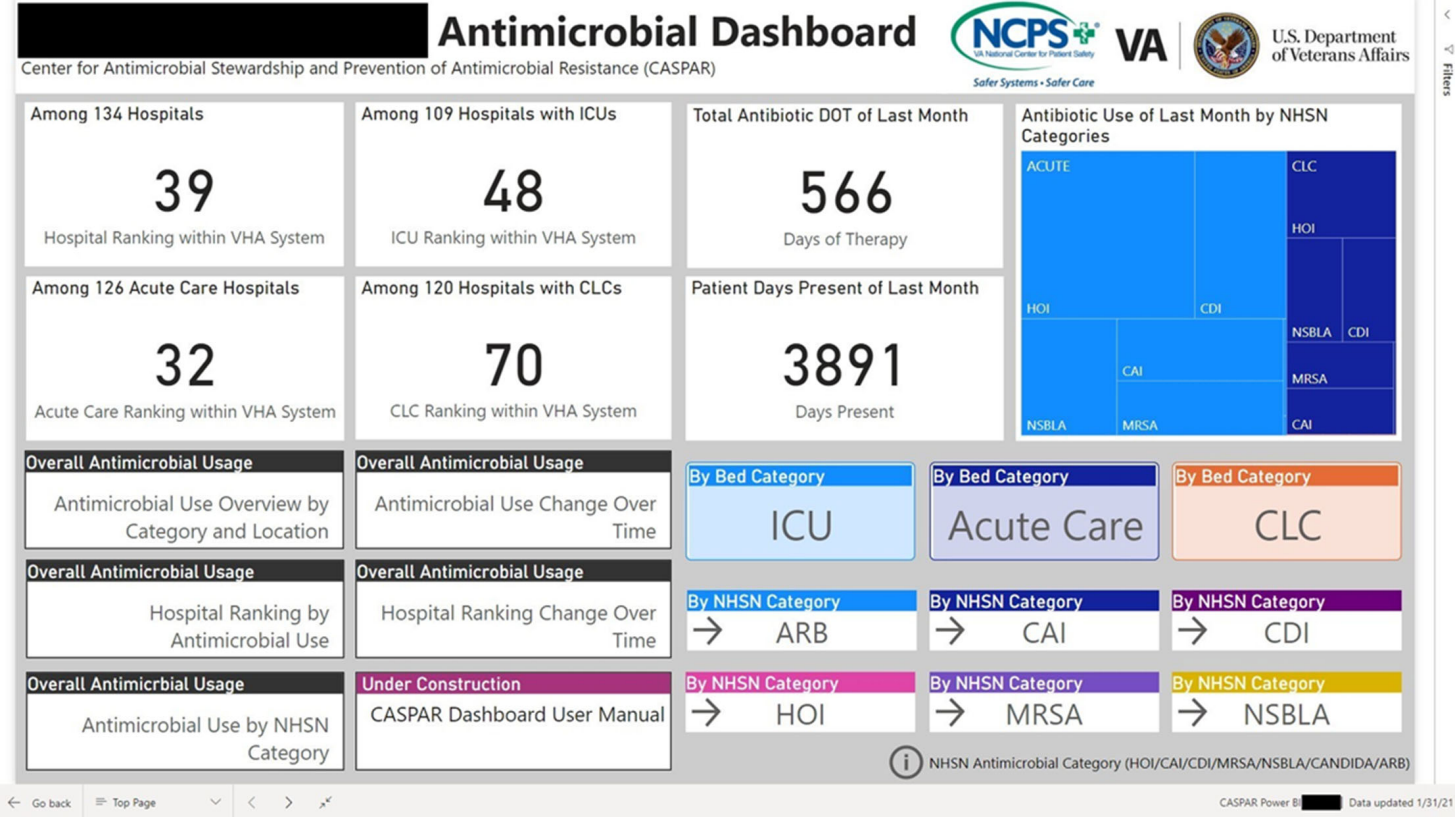

**Figure 2.**
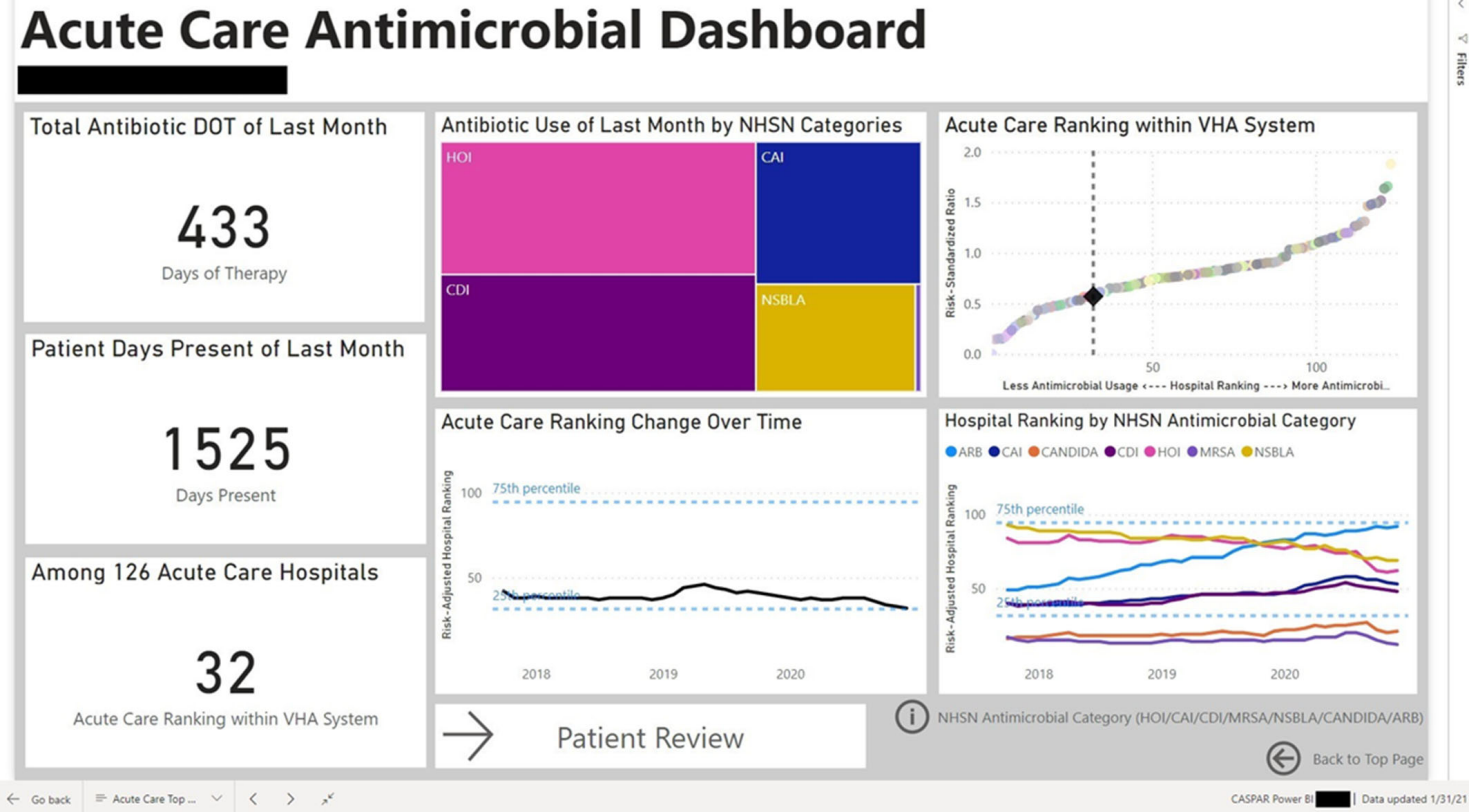

**Figure 3.**